# Knowledge and involvement of husbands in maternal and newborn health in rural Bangladesh

**DOI:** 10.1186/s12884-018-1882-2

**Published:** 2018-06-18

**Authors:** Ahmed Ehsanur Rahman, Janet Perkins, Sajia Islam, Abu Bakkar Siddique, Md Moinuddin, Mohammed Rashidul Anwar, Tapas Mazumder, Adnan Ansar, Mohammad Masudur Rahman, Shahreen Raihana, Cecilia Capello, Carlo Santarelli, Shams El Arifeen, Dewan Md Emdadul Hoque

**Affiliations:** 1 International Centre for Diarrhoeal Disease Research, Bangladesh (icddr,b), Mohakhali, Dhaka, 1212 Bangladesh; 2 Enfants du Monde (EdM), Route de Ferney 150, Geneva, Switzerland

**Keywords:** Male involvement, Maternal and newborn health, Birth preparedness and complication readiness, Skilled birth attendance, Antenatal care, Postnatal care

## Abstract

**Background:**

Access to skilled health services during pregnancy, childbirth and postnatal period for obstetric care is one of the strongest determinants of maternal and newborn health (MNH) outcomes. In many countries, husbands are key decision-makers in households, effectively determining women’s access to health services. We examined husbands’ knowledge and involvement regarding MNH issues in rural Bangladesh, and how their involvement is related to women receiving MNH services from trained providers.

**Methods:**

We conducted a cross-sectional survey in two rural sub-districts of Bangladesh in 2014 adopting a stratified cluster sampling technique. Women with a recent birth history and their husbands were interviewed separately with a structured questionnaire. A total of 317 wife-husband dyads were interviewed. The associations between husbands accompanying their wives as explanatory variables and utilization of skilled services as outcome variables were assessed using multiple logistic regression analyses.

**Results:**

In terms of MNH knowledge, two-thirds of husbands were aware that women have special rights related to pregnancy and childbirth and one-quarter could mention three or more pregnancy-, birth- and postpartum-related danger signs. With regard to MNH practice, approximately three-quarters of husbands discussed birth preparedness and complication readiness with their wives. Only 12% and 21% were involved in identifying a potential blood donor and arranging transportation, respectively. Among women who attended antenatal care (ANC), 47% were accompanied by their husbands. Around half of the husbands were present at the birthplace during birth. Of the 22% women who received postpartum care (PNC), 67% were accompanied by their husbands. Husbands accompanying their wives was positively associated with women receiving ANC from a medically trained provider (AOR 4.5, *p* < .01), birth at a health facility (AOR 1.5, *p* < .05), receiving PNC from a medically trained provider (AOR 48.8, *p <* .01) and seeking care from medically trained providers for obstetric complications (AOR 3.0, *p* < 0.5).

**Conclusion:**

Husbands accompanying women when receiving health services is positively correlated with women’s use of skilled MNH services. Special initiatives should be taken for encouraging husbands to accompany their wives while availing MNH services. These initiatives should aim to increase men’s awareness regarding MNH issues, but should not be limited to this.

**Electronic supplementary material:**

The online version of this article (10.1186/s12884-018-1882-2) contains supplementary material, which is available to authorized users.

## Background

The mid-1990s marked an important turning point internationally in the dialogue regarding the roles and responsibilities of men in reproductive, maternal, newborn and child health (RMNCH) [1, 2]. Prior to this time, relevant policies, strategies and programs almost exclusively focused on women and promoted their roles in improving RMNCH. The Cairo International Conference on Population and Development (ICPD), held in 1994, was the first global initiative that urged for extending the focus beyond women and emphasized the shared responsibility of men in RMNCH. The ICPD Program of Action explicitly called on policy-makers and programmers to engage men in RMNCH actions, including promoting their active involvement in parenthood and family planning [3]. Subsequently, more initiatives began to target men in programs related to reproductive health [4–8]. More recently, increased emphasis has been placed on the involvement of men in maternal and newborn health (MNH) [5]. Notably, the World Health Organization (WHO) issued a recommendation in 2015 encouraging interventions aiming to promote the involvement and engagement of men in MNH for improving care of women within the home during and following pregnancy and for increasing the utilization of skilled MNH services [9].

In patriarchal societies, pregnancy and childbirth are often regarded as women’s exclusive concerns. At the same time, men, typically husbands, act as the major gatekeepers and primary decision-makers within households, effectively determining the care-seeking practices of women during the perinatal period [10–13]. Therefore, involving husbands and men in decision-making processes can play a crucial role in reducing the ‘three delays’ (i.e. the delay in deciding to seek care, the delay in reaching health services, and the delay in receiving adequate and appropriate treatment once at a health facility), thereby facilitating women’s access to and utilization of skilled MNH services [14–16]. In many conservative societies, knowledge, awareness, and participation of men in MNH issues is inadequate and/or inappropriate which negatively impacts on the health of women and newborns [14, 17–20]. Appropriately leveraging the roles of men to positively influence decision-making around MNH is therefore an important avenue for improving the health and wellbeing of women and newborns, with the caveat that such actions toward these ends do not reinforce unequal power dynamics or run counter to the preferences and desires of women.

High coverage and quality of antenatal care (ANC), postnatal care (PNC), and skilled attendance at birth are major drivers for improving MNH and averting avoidable maternal and newborn deaths [21–23]. The remarkable progress of Bangladesh in reducing the maternal mortality ratio (MMR) in the past two decades is largely attributable to the expanded availability and accessibility of these important MNH services [24, 25]. However, the nationwide coverage of key MNH services remains critically low, as only 31% pregnant women attend at least four ANC visits, 37% of births occur in health facilities and 36% women receive PNC from a medically trained provider within the first two days after birth [26]. Only 5% of women who give birth outside of a health facility receive PNC from a medically trained provider within the first two days following birth [26]. These figures clearly demonstrate that the potential for improving MNH through increasing utilisation of these key services has not been fully leveraged to date and remains one of the keys to advancing the survival and thriving of women and newborns throughout the country. Multiple factors operating at each level of delay in the three-delays model contribute to low utilization of skilled MNH services. Increasing the utilization of these services in Bangladesh will require a comprehensive approach, including efforts to expand the availability of quality health services, while simultaneous taking action to increase demand for and access to these services. Promoting the involvement of husbands is a particularly promising strategy for overcoming the first two delays: the delay in deciding to seek care and the delay in reaching services once the decision has been made.

Appropriately and effectively promoting the involvement of men in MNH requires an understanding of the current status of male involvement. A qualitative exploration among women and men of Bangladesh revealed that there are gaps in husbands’ knowledge and involvement regarding maternal health issues [27]. To date, the research conducted in Bangladesh on male involvement has been primarily qualitative in nature, and therefore unable to quantify the gaps in knowledge, awareness and participation of men in MNH issues and their relationship with utilization of MNH services and outcomes.

The Government of Bangladesh, in collaboration with PARI Development Trust, a local non-governmental organization (NGO), has been implementing a health promotion program in selected areas of a rural sub-district of Netrokona district in Bangladesh since 2008. The health promotion program is based on the WHO framework for Working with Individuals, Families and Communities (IFC) to improve MNH, which fosters the implementation of community engagement/health promotion interventions. Enfants du Monde (EdM), an international NGO based in Geneva, Switzerland has been providing technical and financial assistance to PARI in implementing the program. In 2014, the program was extended to new areas within the same sub-district and the International Centre for Diarrhoeal Disease Research, Bangladesh (icddr,b) was included as partner to evaluate the effectiveness of the program focusing on the new areas. In addition to evaluating the effectiveness over time, this expansion provided an opportunity to examine the existing MNH situation through a baseline survey and explore the involvement of men in MNH. In this article, we examine the current status of knowledge and practices of husbands regarding MNH issues, explore how husband’s knowledge is associated with their involvement in MNH, defined in terms of accompanying their wives when receiving MNH services, and finally assess the correlation between their involvement and women’s utilization of skilled MNH services.

## Methods

### Study design

A quasi-experimental study was designed to evaluate the effect of the implementation of the health promotion program based on the WHO recommended IFC framework on MNH-related knowledge and practices. As a part of this study, a cross-sectional survey was conducted in the intervention site and a comparison site in 2014 as the baseline. We present here select findings from the baseline survey where husbands’ knowledge and involvement in MNH was assessed as an exploratory component.

### Study settings

The health promotion program was implemented in the Kalmakanda sub-district of Netrokona district in Bangladesh with a total population of around 180,000 [28]. The intervention sub-distirct was pre-selected in consultation with the Government of Bangladesh and on account of operational efficiency as the implementing NGO (PARI) was based in that sub-district. An adjacent sub-district (Barhatta) with a total population of around 272,000 [28] was selected as the comparison site where there was no large scale MNH initiative in place. Netrokona was selected as an intervention site as it was among the 14 lowest performing districts of Bangladesh with low coverage of MNH specific interventions, and high newborn and child mortality rates [28, 29].

### Study population

Eligible respondents were married women between 19 and 49 years of age with a birth history in the 12-month period preceding the date of survey and husbands of these women. Women with a history of abortion during the 12 months preceding the survey and their husbands were excluded from the study to avoid any potential psychological trauma to the respondents by recalling the events associated with it.

### Sample size

This study was embedded in another larger study which was designed to evaluate the effect of the health promotion program between baseline and endline. As a part of the larger study, we interviewed 725 women with a recent birth history at baseline (444 from the intervention site and 281 from the comparison site). As a part of an exploratory component, we approached all husbands of these women and conducted 317 interviews successfully. Information from 317 wife-husband dyads are presented in this paper.

### Sampling

We adopted a multi-stage cluster sampling technique to select the eligible respondents. In the first stage, four unions were randomly selected from each of the selected sub-districts (intervention and comparison). Unions are the smallest administrative unit of Bangladesh with an approximate population size of 25,000–30,000. In the second stage, four clusters (average population of approximately 1000) were selected from each of the comparison unions and six clusters were selected from each of the intervention unions using the probability proportional to size (PPS) sampling technique. All eligible respondents were included from the selected clusters.

### Data collection

The household survey was conducted through interviewer-administered questionnaires with structured questions. The questions were adapted from the Bangladesh Demographic and Health Survey (BDHS) 2011 and Bangladesh Maternal Mortality Survey (BMMS) 2010 [25, 30]. Separate questionnaires were developed for women and their husbands. The questionnaire started with questions regarding personal and socioeconomic information followed by questions related to knowledge and practices surrounding pregnancy and childbirth. Most of the questions regarding knowledge and care-seeking practice surrounding pregnancy and childbirth were the same for both women and their husbands. However, the husband’s questionnaire had additional questions on whether they accompanied their wives when receiving MNH services. The questionnaires were pre-tested on women with a recent birth history and their husbands residing in non-selected clusters of the selected unions. Interviewers were recruited locally so that they would be familiar with the local context, culture and dialect. Adequate training of the data collectors was ensured by experienced trainers and field supervisors. In the first stage of data collection, a sketch map was drawn for each of the selected clusters indicating boundary, landmark and Bari (extended household) locations. All households and women who had a birth outcome in the 12 months preceding the survey were enumerated and listed. In the second stage of data collection, all eligible women were interviewed with the structured questionnaire. The husbands of these women were interviewed after their wives separately by another team of data collectors. The interviewers who interviewed the wives did not disclose whether their husbands would be interviewed.

### Data analysis

Data analysis was conducted using the Stata V.13 (StataCorp. 2013. Stata Statistical Software: Release 13. College Station, TX: StataCorp LP.)

Husbands’ education, women’s education and women’s age were converted into categorical variables. Due to small numbers, all other religions except Muslim were grouped into one category and coded as “other”. Household level variables such as household possessions; materials used for construction of the floor, wall, and roof; drinking water source; toilet facilities; and ownership of land and domestic animals were used to generate socio-economic indices through principal component analysis. Based on the socio-economic indices of the households that we have interviewed, the wealth quintile was generated [31, 32] (Table 1).

**Table 1:** Background characteristics of women and their husbands (*N* = 317)

Background characteristics	Wife (*N* = 317)	Husband (*N* = 317)
	%	%
Age		
15–24 years	41.0	–
25–44 years	49.5	–
45+ years	9.5	–
Mean age in years (SD)	26.5 (±5.7)	–
Education		
Primary incomplete (0–4 years)	56.8	66.6
Primary complete to secondary incomplete (5–9 years)	37.2	28.4
Secondary complete or higher (10+ years)	6.0	5.0
Mean years of schooling (SD)	3.9 (±3.5)	3.0 (±3.6)
Involvement in income generating activities	3.2	NA
Religion		
Muslim	91.8	91.8
Others (Hindu/Christian etc.)	8.2	8.2
Wealth quintile		
Lowest	20.8	
Second	18.3	
Middle	18.6	
Fourth	18.9	
Highest	23.3	

In addition to the socio-demographic characteristics of respondents, measures of knowledge and practices related to pregnancy and childbirth and husbands’ involvement in MNH were also assessed. Categories of knowledge included awareness of women’s rights related to MNH; knowledge of danger signs during pregnancy, birth and following birth; and awareness of the need to seek skilled MNH services. Categories of practices included birth preparedness and complication readiness (BPCR) and utilization of skilled MNH services (ANC, delivery, PNC and care obstetric complications). Husbands’ involvement was defined as husbands accompanying their wives to a health facility or a health care provider for receiving a particular MNH service, i.e. ANC, childbirth, PNC and care-seeking for obstetric complications, and staying at the site of the facility or with their wives while they received the service. According to this definition, husbands could either be in the room with the woman while receiving the service or at another location within the premises where the service was received.

The difference between women’s knowledge and their husbands’ knowledge was assessed using proportion test (z test). The associations of husbands’ knowledge with their involvement were assessed using multiple logistic regression where husbands’ knowledge was considered as an explanatory variable and husbands’ involvement was considered as an outcome variable.

Women receiving ANC, skilled care at birth, PNC and care for obstetric complications from trained health care providers were considered as outcome variables. Involvement of the husband (i.e. husbands present with their wives while receiving MNH services) was considered as the primary explanatory variable. Separate multiple logistic regression models were used to measure the effect of husbands’ involvement on each of the outcome variables (i.e., receiving ANC, skilled care at birth, PNC and care for obstetric complications from trained health care providers).

For all multiple logistic regression models reported in this paper, we controlled the effect of all confounders while presenting the associations between the primary exposure variable and outcome of interest. A covariate was considered as a confounder if it was significantly associated with both the primary exposure variable and outcome of interest in the binary logistic regression.

### Ethical approval and consent

Ethical approval for the study was obtained from the Ethical Review Committee (Federal Wide Assurance #00001468) of the International Centre for Diarrhoeal Disease Research, Bangladesh (icddr,b). Informed and written consent was collected from all participants prior to the interviews.

## Results

Table 1 presents the socio-demographic characteristics of women and their husbands. Around half of the women had no education or less than four years of schooling, and only 6% had more than 10 years of schooling. A negligible proportion (3%) of the women was involved in income-generating activities. Around two-thirds of the husbands had no education or less than four years of schooling, and only 5% had more than 10 years of schooling. The majority of respondents (92%) were Muslim. Regarding household possessions, nearly 80% owned a mobile phone and only 14% had a television in their homes. Among the wife-husbands dyads, 20% were in the lowest-wealth quintile and 23% in the highest.

**Fig. 1:**
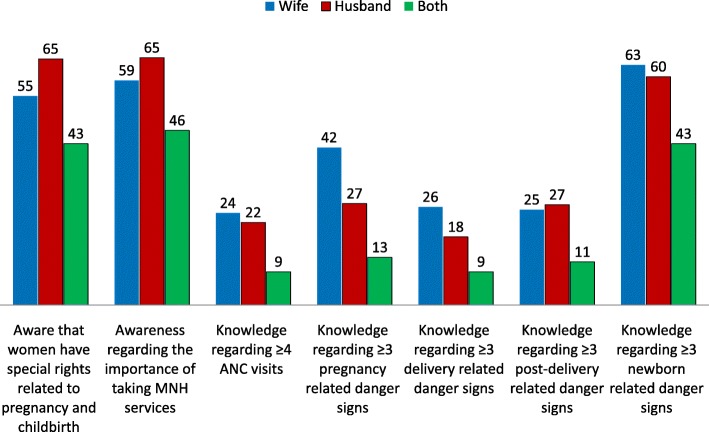
Percentage distribution of knowledge and awareness of women and their husbands regarding MNH issues (*N* = 317)

Figure 1 presents the level of knowledge of women and their husbands regarding MNH issues. Sixty-five percent of husbands reported that they were aware that women have special rights related to pregnancy and childbirth, which was significantly (*p* = 0.012) higher than that of women (55%). Around two-thirds of husbands reported that they were aware of the importance of women availing MNH services. Only 22% husbands were aware of the importance of women attending at least four ANC contacts during pregnancy. Around one-quarter of men could mention three or more pregnancy-related danger signs, which was significantly (*p* = 0.001) less than the corresponding knowledge level of women (42%). Less than one-fifth of husbands could recall three or more delivery-related danger signs. Approximately two-thirds of women and their husbands could mention at least three newborn-related danger signs. Only 7% of the husbands could mention three or more danger signs related to pregnancy, birth, postpartum and newborns.

Knowledge of women was significantly associated with the knowledge of their husbands with respect to the awareness of rights of women related to MNH, availability of MNH services, importance of attending at least four ANC contacts, as well as awareness of at least three danger signs in all categories (during pregnancy, birth, postpartum and in newborns) (*p* < 0.05).

**Fig. 2:**
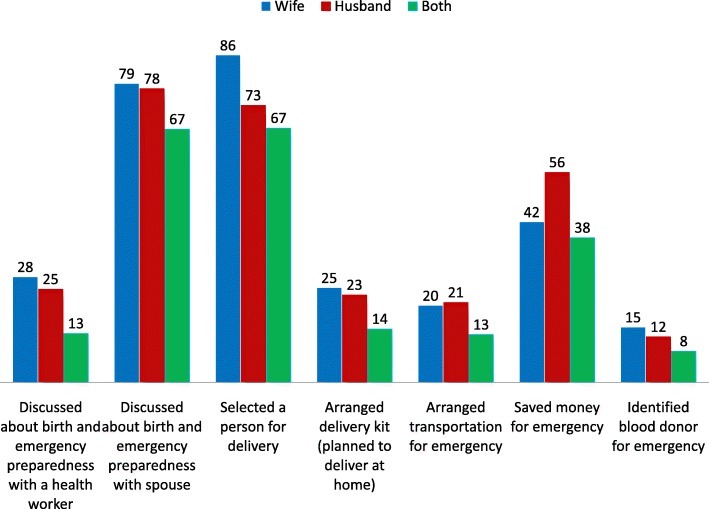
Percentage distribution of engagement of women and their husbands in BPCR practice (*N* = 317)

Figure 2 shows the engagement of women and their husbands in BPCR practice. Around one-fifth of the husbands did not discuss BPCR with their wives during pregnancy. Only a quarter reported having had discussions around BPCR with a health care provider. Three-quarters reported having been involved in selecting a birth place or a person to attend the birth in advance and over half reported that they saved money in advance for potential obstetric emergencies. However, only 21% husbands arranged transportation and 12% identified a potential blood donor for obstetric emergencies. Women’s engagement in BPCR was similar to their husbands’ across most of the categories. However, more women reported having selected a birth place or birth attendant than their husbands (86% vs 73%, *p* < 0.001). On the contrary, fewer women reported having saved money for obstetric emergencies (42% vs 56%, *p <* 0.001).

**Fig. 3:**
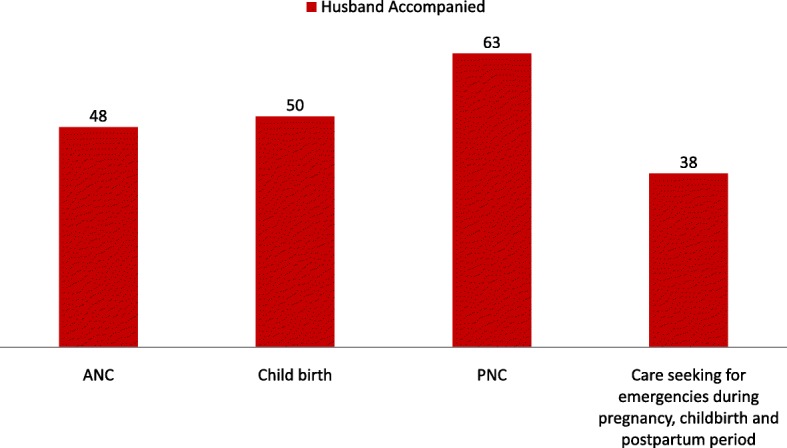
Percentage distribution of women accompanied by their husbands while receiving MNH services

Figure 3 presents the level of involvement of husbands in terms of accompanying their wives while receiving different MNH services. Among women who attended any ANC during their most recent pregnancies, around 48% were accompanied by their husbands. Around half of the husbands were physically present at the birthplace during the time of delivery. Approximately two-thirds of women were accompanied by their husbands while receiving PNC. Only one-third of the husbands accompanied their wives while seeking care for emergencies during pregnancy, childbirth and the postpartum period.

Table 2 illustrates the association between husbands’ knowledge and accompanying their wives while receiving MNH services. Knowledge regarding importance of women attending at least four ANC contacts during pregnancy was significantly associated with husbands being physically present at the birth place during childbirth (AOR 1.8; CI 1.1–2.9) and accompanying women while receiving PNC services (AOR 2.5; CI 1.2–5.1). Husbands’ awareness of at least three pregnancy-related danger signs was associated with their accompanying their wives while receiving ANC services (AOR 1.7; CI 1.01–3.0). A similar correlation was observed between husbands’ awareness of at least three newborn danger signs and presence during ANC (AOR 1.9; CI 1.1–3.3).

**Table 2:** Association between husbands’ knowledge and accompanying their wives while receiving MNH services (*N* = 317)

Husbands’ Knowledge and Awareness	Husbands accompanying their wives while receiving ANC		Husbands physical presence during child birth		Husbands accompanying their wives while receiving PNC	
	OR (CI)	AOR (CI)	OR (CI)	AOR (CI)	OR (CI)	AOR (CI)
Awareness that women have special rights related to pregnancy and childbirth	1.4 (0.8–2.6)	1.3 (0.8–2.3)	1.9* (1.2–3.1)	1.9 (0.9–3.7)	1.7 (0.8–3.9)	1.5 (0.7–3.1)
Awareness regarding the importance of receiving MNH services	1.5 (0.9–2.8)	1.4 (0.8–2.4)	1.8* (1.1–3.0)	1.7 (0.8–3.3)	1.7 (0.8–3.8)	1.3(0.6–2.8)
Knowledge regarding ≥4 ANC visits	1.6 (0.9–2.7)	1.4 (0.8–2.4)	1.9* (1.2–3.0)	1.8* (1.1–2.9)	3.4* (1.7–7.3)	2.5* (1.2–5.1)
Knowledge regarding ≥3 pregnancy related danger signs	1.6 (0.9–2.8)	1.7* (1.01–3.0)	1.4 (0.8–2.5)	1.6 (0.8–3.4)	1.1 (0.5–2.3)	1.1 (0.5–2.3)
Knowledge regarding ≥3 delivery related danger signs	1.6 (0.8–3.0)	1.4 (0.7–2.6)	1.5 (0.8–2.8)	0.9 (0.4–2.1)	2.8* (1.3–5.8)	2.3* (1.1–4.8)
Knowledge regarding ≥3 post-delivery related danger signs	1.4 (0.8–2.5)	1.2 (0.7–2.2)	0.9 (0.5–1.5)	0.7 (0.4–1.2)	1.3 (0.6–2.7)	1.1 (0.5–2.2)
Knowledge regarding ≥3 newborn related danger signs	1.9* (1.1–3.4)	1.9* (1.1–3.3)	1.1 (0.7–1.8)	1.3 (0.7–2.5)	1.1 (0.6–2.2)	1.1 (0.6–2.2)

*p < 0.05

Table 3 presents the association relation between husbands accompanying their wives when receiving MNH services and women’s overall utilization of skilled MNH services. Husbands accompanying their wives to a health facility for ANC was associated with women receiving ANC from a medically trained provider (AOR 4.5, *p* = 0.001). Similarly, women had twice the odds of giving birth in a health facility when their husbands were present at the facility during the time of childbirth (OR 2.0, *p* = 0.027; AOR 1.5, *p* = 0.220). Similarly, women whose husbands accompanied them to receive PNC were more likely to have received PNC from a medically trained provider compared to women who were not accompanied (AOR 48.8, *p* = 0.000). The probability of women receiving care from a qualified provider in response to obstetric emergencies was three times higher when the women were accompanied by their husbands (AOR 3.0, *p* = 0.018).

**Table 3:** Association between husbands’ accompanying their wives and utilization of skilled MNH services

Type of MNH services	Husbands accompanying their wives				
	Total %	Yes %	No %	Crude OR (CI)	Adjusted OR (CI)
ANC	(*N* = 181)	(*N* = 86)	(*N* = 95)		
Unqualified provider	42.0	23.3	58.9	4.7*(2.5-9.0)	4.5*(2.3-8.7)
Qualified provider	58.0	76.7	41.1		
Birth	(*N* = 317)	(*N* = 158)	(*N* = 159)		
Home	84.9	80.4	89.3	2.0*(1.1-3.9)	1.5(0.8–3.1)
Health facility	15.1	19.6	10.7		
PNC	(*N* = 71)	(*N* = 45)	(*N* = 26)		
Unqualified provider or Qualified provider (> 2 days)	29.6	4.4	73..1	58.4*(11.1-307.4)	48.8*(8.17-291.4)
Qualified provider in ≤2 days	70.4	95.6	26.9		
Care-seeking for complications during pregnancy, delivery and after delivery	(*N* = 112)	(*N* = 42)	(*N* = 70)		
Unqualified provider	76.8	64.3	84.3	3.0* (1.2–7.3)	3.0* (1.2–7.3)
Qualified provider	23.2	35.7	15.7		

*p < 0.05

## Discussion

In passing from the era of the Millennium Development Goals (MDG) to the United Nations 2030 Agenda for Sustainable Development, the international community has established the Sustainable Development Goals (SDGs) and set the target for countries to reduce maternal mortality ratio to less than 70 per 100,000 live births by 2030 [33]. Bangladesh has already declared its commitment to achieve the SDG targets. The new Maternal Health Strategy of Bangladesh, which was published in 2015, envisions reaching the 2030 SDG target by achieving very high national coverage of skilled MNH services [34]. This implies that Bangladesh will need to significantly accelerate the annual rate of increase of coverage of skilled MNH services in the SDG era from what was observed during the MDG period between 1990 and 2015 [25, 26, 30]. Therefore, Bangladesh needs to go beyond its usual practice and adopt innovative approaches to increase the coverage of skilled MNH services to reach the ambitious SDG targets. Expanding initiatives to include husbands and promoting their involvement in MNH could be considered as an important strategy for adaptation in this regard.

This study demonstrates that husbands’ involvement is a key factor associated with women’s utilization of skilled MNH services during pregnancy, childbirth and postpartum in rural Bangladesh. Specifically, we found that women were at increased odds of receiving skilled MNH services when they were accompanied by their husbands. This correlation remained consistent across all categories for both routine and emergency obstetric care. However, while women were at increased odds of giving birth at a facility when their husbands were present at the birthplace, this relationship was not significant after adjustment for confounding factors.

The global evidence on the association between male involvement in MNH and women’s use of skilled MNH services has been mixed, with studies in different settings finding varying effects of male involvement depending on the type of service [13, 35, 36]. In Nepal, involving men in ANC education was associated with increased use of PNC, though the same results were not observed for ANC or skilled birth attendance [14]. In India [19] and Tanzania [37], male involvement has been associated with an increase in skilled attendance at birth. However, studies related to male involvement have typically been conducted within the context of testing interventions, and often a package of interventions. The findings of this study suggest that the presence of men in and of itself is associated with of use of skilled services, providing justification for interventions which aim to promote the participation of men in MNH services [25, 26, 30, 33, 34].

The findings of this study suggest that knowledge and awareness of husbands regarding rights related to MNH, the importance of availing MNH services and danger signs related to pregnancy, childbirth and the postpartum period remains low in rural Netrokona. This is similar to the findings reported by other studies conducted in Southeast Asia and Africa [4, 16, 27], though there are some notable exceptions [38, 39]. We found that husbands have a greater awareness of newborn-related danger signs compared to maternal danger signs. This may be explained by cultural norms in which the health of the infant is prioritized to a greater degree than that of the woman [40, 41]. It could also be explained by the fact that adverse newborn outcomes (i.e. newborn mortality and morbidity) are far more common than adverse maternal outcomes and therefore men are more likely to be aware of experiences regarding newborn complications than maternal health complications. Another explanation for this difference could be that in patriarchal societies maternal health is regarded as women’s exclusive health concern, thus translating into poor levels of knowledge of husbands regarding maternal health issues [10, 42, 43]. In any case, it would be worth examining this in more depth as it may have possible programmatic implications in terms of using newborn health as an entry point for increased prioritization of women’s health during pregnancy and childbirth.

In this study, the correlation between knowledge and involvement of husbands in accompanying women to MNH services was more difficult to establish. Indeed, after adjusting for confounders, we only found statistical significance in the relationship between the level of knowledge of men and the likelihood of their accompanying women during MNH services in certain categories. However, there was a clear trend between knowledge of husbands regarding MNH issues and their involvement in utilization of relevant services as the odds ratios were universally greater than one for all categories. We believe that the statistical significance was not achieved for most of the categories due to our small sample size. Findings from other studies have been mixed, with studies in Nepal [14] and Uganda establishing a positive correlation between men’s knowledge and participation in MNH services [15], and another study in Nigeria failing to find this correlation [44]. This is an important finding with programmatic implications. Investing in the knowledge and awareness of husbands has the potential of increasing the involvement of husbands in MNH issues, which in turn may increase the utilization of skilled services.

However, the findings of this study would suggest that focusing exclusively on knowledge is likely insufficient. Effectively intervening to promote male involvement may require expanding the focus beyond actions aiming to increase knowledge of men, for example by addressing cultural and structural barriers to male involvement. Factors which have been identified in previous studies as barriers include social stigma, shyness and embarrassment, lack of men’s time availability due to job responsibilities, and structural issues within the health services such as the service availability, readiness and accessibility of health facilities, maternal health services which are not male-friendly and hospital policy restrictions [36, 45, 46]. Promotion of male-friendly services and issuing of invitation letters have been proposed as potential interventions to address certain structural barriers to male involvement, and have demonstrated some promise [4, 47].

WHO recommends BPCR as one of the important interventions for increasing utilization of skilled MNH services and thus averting avoidable maternal deaths [9, 48]. We have identified critical gaps in BPCR for both women and their husbands in this study which is consistent with the findings from other studies conducted in Bangladesh and low-income countries [49–52]. However, in this study, husbands’ participation in elaborating the plan appears to be somewhat selective, as they are involved primarily in selecting a birth place or attendant and saving money for potential obstetric emergencies. Globally, the involvement of husbands in BPCR appears to be context specific, with low involvement overall in some regions [53, 54] and high in others, [55] and selective across components in all cases. This would suggest that the discriminate involvement of husband in BPCR in Netrokona may be due to the conservative cultural context, which could also explain the lower percentage of women involved in saving money or identifying a potential blood donor for emergencies.

It is worth noting that within all categories, women’s knowledge of danger signs is significantly correlated with husbands’ knowledge of danger signs. A similar trend was noticed in BPCR practice. The study was not designed to assess the direction or the temporal relationship of these associations but may indicate the importance of promoting dialogue within the family regarding MNH issues. This should be explored in more detail as dialogue is a necessary precursor to shared decision-making, which is not only important for women’s empowerment, but has also been linked to positive care-seeking behaviour in Bangladesh [56].

Based on the study findings, we recommend that programs and initiatives consider involving husbands in MNH issues. Investing in the knowledge and awareness of husbands can be adopted as one avenue for increasing their involvement in MNH, though it is important to also assess and address other cultural and structural factors which serve to impede male participation in MNH.

In all cases it is important to carefully plan such interventions and approach them with caution, particularly as promoting the engagement of men in MNH can lead to negative unintended consequences, potentially running in direct conflict to women’s empowerment and decision-making, and reinforcing unbalanced power dynamics related to gender [12, 57]. It is also critical to explore the desires of women regarding their husbands’ involvement. Indeed, some studies have found that women do not want their husbands to be involved in MNH as this infringes on areas of their lives in which they currently have autonomy and are able to engage with others outside their households [58]. Efforts to engage men in MNH should be coherent with the expectations and desires of women and not be implemented to the detriment of their autonomy and decision-making.

Along this vein, it is also important to better understand husbands’ motivations and preferences for involvement in MNH. Studies to date have been mixed as to whether husbands wish to be involved in maternal health [18, 59]. However, it is worth noting that these studies have been conducted primarily within the African context. Greater understanding regarding the underlying gender relations and how involvement of husbands can lead to gender-transformative change with regard to MNH is imperative for effectively designing MNH interventions promoting husbands’ involvement, particularly in patriarchal structures [11].

We recommend future research to better understand women’s desires with regard to male involvement and investigate the desires and motivations of husbands to be involved in MNH issues in the context of Bangladesh and South Asia. Only through a deeper understanding of the gender dynamics will it be possible to develop programmes and policies that move beyond an instrumentalist approach to the involvement of men toward gender-transformative approaches.

One of the limitations of this study is its generalisability. The survey was conducted in Netrokona which is one of the disadvantaged and lowest performing districts in Bangladesh [29]. The socio-demographic profile of the study sample also diverges in important ways from national rural estimates. For example, nearly half of the male respondents never attended school compared to the national rural estimate of 23% [26]. As for household possessions, a lower percentage of the study population possessed a television (14%) compared to the national rural estimate of 33%. Moreover, a much lower percentage of the female respondents reported being involved in income generating activities (3.2%) compared to women living in rural areas nationally (24%) [26]. Utilization of key MNH services remains alarmingly low among the study population. Only 58% of female respondents in the sample received any ANC during their most recent pregnancy which is much lower than the national rural estimate (75%) [26]. Similarly, only 15% of our female respondents gave birth in a health facility compared to 31% of rural women nationally. Regarding PNC there is also a wide gap between our sample estimate and national rural estimate (22% vs 64%) [26]. These figures reflect the poor socio-demographic situation, as well as gaps in utilization of MNH services in rural areas of Netrokona district. The figures also call for an urgent need to identify effective strategies to increase utilization MNH services and improve the health of mothers and newborns in low performing and disadvantaged districts like Netrokona.

Another limitation of this study is that we were able to capture husbands’ involvement in BPCR and in accompanying women when seeking MNH services, but did not capture other areas in which men can be involved in MNH, such as in supporting women in self-care within the household or involvement in newborn care. Further research should be conducted to better understand cultural and personal preferences in order to establish the best measures of male involvement in MNH in the Bangladeshi context.

The high non-response rate of husbands is also a limitation of the study. Despite repeated attempts (three visits on three separate days) by the data collectors, a significant proportion of the husbands could not be reached as they were not available at their households during the visit or not residing the study site for professional reasons. However, we have compared the background characteristics of the wives whose husbands were interviewed with those whose husbands could not be interviewed. No significant difference was noted between these two groups (Additional file 1: Table S1). Therefore, we believe that the high non-response rate of husbands did not affect our study results.

Another limitation of this study is the possible recall bias, as we were asking question related to the past, even if not so distant. It is quite possible that respondents were not able to accurately remember the details which they were asked about. However, the recall period in our survey related to utilization of MNH services was much smaller than that of other standard surveys [25, 26]. There is also the possibility of social desirability bias, though we tried to limit this by recruiting local data collectors.

Moreover, as the husbands were interviewed at a different time from women by a separate team of data collectors, we are not sure whether the husbands had received information regarding the questionnaire from their wives prior to the interviews. However, the likelihood is very slim. The interviewers who interviewed the wives did not disclose whether their husbands would be interviewed. In addition, the questionnaire was quite long, and it is highly unlikely that the wives would be able to remember the entire questionnaire and share the information with their husbands.

Finally, we have presented the findings from a cross-sectional survey in this paper. Therefore, we were limited with our scope to draw causal inference between the explanatory and outcome variables.

## Conclusion

Achieving increased access to and utilization of routine and emergency obstetric and neonatal health services is central to ensuring that women and newborns are able to enjoy optimal health and realize their rights. While the recognition of the important role of men in improving MNH has gained significant momentum, the evidence remains limited regarding how to best involve them to improve the health and wellbeing of women and newborns and the impact of their involvement. Our findings suggest that husbands’ involvement is positively correlated with women’s utilization of skilled MNH services in rural Bangladesh, leading us to conclude that strategies to engage husbands in MNH should be prioritized for achieving the 2030 SDG targets.

## Additional file

Additional file 1: Summary of background characteristics. (DOCX 15 kb)

## Abbreviations

ANC: Antenatal Care
BPCR: Birth Preparedness and Complication Readiness
CI: Confidence Interval
icddr,b: International Centre for Diarrhoeal Disease Research, Bangladesh
ICPD: International Conference on Population and Development
IFC: Individuals, Families and Communities
IRB: Institutional Review Board
MMR: Maternal Mortality Ratio
MNH: Maternal and Newborn Health
NGO: Non-governmental Organization
PNC: Postnatal Care
RMNCH: Reproductive, Maternal, Newborn and Child Health
WHO: World Health Organization
