# Supplementary material for: Knowledge and involvement of husbands in maternal and newborn health in rural Bangladesh
Source: BMC Pregnancy Childbirth. 2018 Jun 18;18:247. doi: 10.1186/s12884-018-1882-2 (PMC6007056; doi:10.1186/s12884-018-1882-2)
Supplement: Supplementary file 1 — Summary of background characteristics. (DOCX 15 kb) [file 12884_2018_1882_MOESM1_ESM.docx]

Table S1: Summary of background characteristics

| **Background characteristics of Women** | **Husbands interviewed (N=317)** | **Husbands could not be interviewed (N=408)** | **p value** |
| --- | --- | --- | --- |
|  | **%** | **%** |  |
| **Women’s age** |  |  |  |
| 15-24 years | 41.0 | 42.9 | 0.607 |
| 25-44 year | 49.5 | 52 | 0.504 |
| 35+ years | 9.5 | 5.1 | 0.022 |
| **Women’s education** |  |  |  |
| Primary incomplete (0-4 years) | 56.8 | 54.4 | 0.519 |
| Primary complete to secondary incomplete (5-9 years) | 37.2 | 40.2 | 0.411 |
| Secondary complete or higher (10+ years) | 6.0 | 5.4 | 0.729 |
| **Husbands’ education** |  |  |  |
| Primary incomplete (0-4 years) | 66.6 | 63.7 | 0.417 |
| Primary complete to secondary incomplete (5-9 years) | 28.4 | 27.7 | 0.835 |
| Secondary complete or higher (10+ years) | 5.0 | 8.1 | 0.099 |
| **Women’s involvement in income generating activities** | 3.2 | 2.9 | 0.815 |
| **Housing possession** |  |  |  |
| Television | 13.9 | 10.8 | 0.205 |
| Mobile | 79.5 | 83.8 | 0.136 |
| **Religion** |  |  |  |
| Muslim | 91.8 | 91.2 | 0.774 |
| Others (Hindu/ Christian etc.) | 8.2 | 8.8 | 0.774 |
| **Wealth quintile** |  |  |  |
| Lowest | 20.8 | 19.4 | 0.640 |
| Second | 18.3 | 19.1 | 0.784 |
| Middle | 18.6 | 23.3 | 0.125 |
| Fourth | 18.9 | 18.9 | 1.000 |
| Highest | 23.3 | 19.4 | 0.202 |
